# Cost Analysis of Diagnostic Endoscopic Procedures for Chronic Diarrhoea

**DOI:** 10.7759/cureus.20039

**Published:** 2021-11-30

**Authors:** Woo Jae Kim, Shadi Abdelrahman, Adam Daneshyar, Ghiath Ismayl, Steve Odogwu

**Affiliations:** 1 Orthopaedics, Russells Hall Hospital, Dudley, GBR; 2 General Surgery, Walsall Manor Hospital, Birmingham, GBR; 3 Orthopaedic Surgery, Russells Hall Hospital, Birmingham, GBR; 4 General Surgery, Walsall Healthcare NHS Trust, Walsall, GBR

**Keywords:** costs and cost analysis, utilization of endoscopy, colonoscopy, general gastroenterology, chronic diarrhoea

## Abstract

Chronic diarrhoea is a common condition that affects up to 5% of the population which heavily affects the quality of life for the patient. The British Society of Gastroenterology guidelines recommend that for those who suffer with chronic diarrhoea, a colonoscopy with a biopsy is recommended to exclude microscopic colitis. This retrospective audit included 147 patients who received endoscopic procedures in 2019 at Walsall Manor Hospital for chronic diarrhoea. The results show that a total of £56,797 was incurred through endoscopic and histological investigation with four patients (2.6%) diagnosed with microscopic colitis. Given the lack of diagnostic yield, there is room for advancement in the current guidelines for managing persistent diarrhoea.

## Introduction

Chronic diarrhoea is defined as loose stools with increased frequency that persists for longer than four weeks and it can affect up to 5% of the population at any given time [1]. There are multiple aetiological causes for chronic diarrhoea such as inflammatory bowel disease, enzymatic deficiencies causing malabsorption or maldigestion, infective causes and irritable bowel syndrome [2]. A surging cause for chronic diarrhoea is microscopic colitis, a form of chronic inflammatory disease of the colon [3]. The spectrum of irritable bowel syndrome and microscopic colitis often overlap clinically and are only differentiated histologically. Two subtypes exist in microscopic colitis: collagenous colitis and lymphocytic colitis [4].

Following initial evaluation with blood markers and stool sample testing, patients often are investigated further endoscopically to find the cause for their persistent diarrhoea [5]. The British Society of Gastroenterology guidelines recommend that for those who suffer with chronic diarrhoea, a colonoscopy with a biopsy is recommended to exclude microscopic colitis [6]. With the current burden on the National Health Service secondary to socio-economic and political factors, it is important to prioritise efficient use of limited resources.

The aim of this retrospective audit is to analyse the costs of performing diagnostic endoscopy and histopathological sampling for patients suffering with chronic diarrhoea within our hospital.

## Materials and methods

Study design

This was a single-centre retrospective audit carried out at Walsall Manor Hospital, a district general hospital. This audit collected data for patients who required endoscopic investigation in 2019 for chronic diarrhoea as the sole symptomatic complaint. There were 300 patients identified who required an endoscopy in 2019 according to the British Society of Gastroenterology guidelines and of those 147 patients were included in our study.

Inclusion criteria

The patient’s sole presenting complaint must be chronic or persistent diarrhoea.

Exclusion criteria

Patients who presented with other symptoms alongside chronic diarrhoea were excluded including per rectal bleeding, acute diarrhoea and altered bowel habit including constipation.

Data collection

Data was collected on an excel spreadsheet and basic analysis tools of excel were utilised to compile the results. Data points included age, presenting complaint, endoscopic diagnosis, biopsy results and interventions following histological diagnosis. Interventions were grouped into symptomatic treatment, no intervention or discharge, medical treatment and surgical treatment.

Costs

Data from the national schedule of NHS costs were used, specifically looking at outpatient procedure costs which are highlighted in Table 1.

**Table 1:** Costs of individual procedure.

Investigation	Cost as outpatient per unit
Colonoscopy + Biopsy +Histology	£412
Flexible Sigmoidoscopy + Biopsy + Histology	£309
Colonoscopy	£239
Flexible Sigmoidoscopy	£199

## Results

The total number of patients included in our study was 147 patients with the vast majority of patients receiving a colonoscopy with a biopsy and histological analysis as highlighted in Table 2. The total cost of the procedures included in our audit was £56,797 with a breakdown of costs shown in Table 3.

**Table 2:** Total number of procedures.

Endoscopic procedure	Total
Colonoscopy + Biopsy +Histology	118
Flexible Sigmoidoscopy + Biopsy + Histology	19
Colonoscopy	8
Flexible Sigmoidoscopy	2

**Table 3:** Total cost of procedures.

Studies	Total cost
Colonoscopy + Biopsy +Histology	£48,616
Flexible Sigmoidoscopy + Biopsy + Histology	£5871
Colonoscopy	£1912
Flexible Sigmoidoscopy	£398
	£56,797

The outcomes of the patients are recorded below in Table 4 which shows that 98 patients were discharged following endoscopic evaluation or received no intervention. A further 29 patients received symptomatic treatment only including medication to slow down gastric transit, stool bulking agents and anti-spasmodic agents. Thirteen patients received medical treatment including four who received immunomodulators for their newly diagnosed microscopic colitis (Table 5) and seven patients received surgical intervention following endoscopy including polypectomies and 1 right hemicolectomy for malignancy.

**Table 4:** Outcomes.

Outcomes	Number of patients
Symptomatic treatment	29
Medical treatment	13
Discharged or no intervention	98
Surgical treatment	7

**Table 5:** Histological findings.

Histology	Number of patients	% of cohort
Collagenous Colitis	2	1.3%
Lymphocytic Colitis	2	1.3%
Nonspecific active colitis	8	5.4%

The total cost of those who were discharged or only received symptomatic management was £48,557 and those who went on to receive medical or surgical management was £8240. Thus, 85.4% of the total cost of procedures did not lead to any significant medical or surgical management, rather symptomatic treatment of the patient (Table 6).

**Table 6:** Cost of outcomes.

Total cost of procedures not leading to surgical or medical management	Total cost of procedures leading to surgical or medical management
£48,557	£8240

## Discussion

From our cohort, four patients (2.6%) were found to have a form of microscopic colitis which is less than the prevalence stated in a recent systematic review which states it is found in 7% of patients who have functional bowel disorders [7]. A possible reason that our audit showed a lower prevalence rate compared to the literature is due to our strict inclusion criteria. Function bowel disorder encompasses multiple facets of symptoms including bloating, abdominal pain, constipation and persistent diarrhoea [8], hence likelihood is that we have inherently removed some of those who may have functional bowel disorder from our study.

A large proportion of our patients who underwent endoscopic investigation were either discharged or treated symptomatically which questions whether the current guidelines for scoping patients with chronic diarrhoea is justified. A systematic review of primary care investigations showed that colonoscopies were one of the investigations overused within the UK primary care healthcare system [9]. A possible reason for this is that a change in bowel habit can signify a significant underlying issue such as inflammatory bowel disease or colorectal cancer [10], thus cannot be ignored. A cost analysis of colorectal cancer screening showed that colonoscopy remained a very cost-effective tool for screening when used every 10 years within a population willing to participate. However, when taken into consideration the population’s willingness to undertake a colonoscopy procedure, it was found that CT-colonography was more cost-effective as a screening tool if provided more than twice in a patient’s lifetime [11].

Furthermore, 85.4% of the total expenditure of our cohort of patients primarily resulted in patients either being discharged or receiving symptomatic management. A recent two-centre study which specifically critiques the guidelines published by the British Society of Gastroenterology found that of the 872 colonoscopies performed, only 1.5% random colonic biopsies yielded the diagnosis of microscopic colitis [12]. The calculated cost per positive diagnosis of microscopic colitis was $10 862.42, leading them to believe that this was not a cost-efficient diagnostic tool. Similar conclusions were reached by Hotouras et al whereby the cost to diagnose two patients with microscopic colitis was £11,028 per patient [13].

## Conclusions

This audit has shown that there is a high expenditure rate of resources in utilising endoscopy as a tool to diagnose the cause for patients with chronic diarrhoea with low diagnostic yield. The national guidelines on managing patients with persistent diarrhoea should be scrutinised, even more so in a period where the National Health Service is under tremendous strain with finite resources.
